# Single-mode termination of phage transcriptions, disclosing bacterial adaptation for facilitated reinitiations

**DOI:** 10.1093/nar/gkae620

**Published:** 2024-07-16

**Authors:** Eunho Song, Sun Han, Heesoo Uhm, Changwon Kang, Sungchul Hohng

**Affiliations:** Department of Physics and Astronomy, and Institute of Applied Physics, Seoul National University, Seoul 08826, Republic of Korea; Department of Physics and Astronomy, and Institute of Applied Physics, Seoul National University, Seoul 08826, Republic of Korea; Department of Physics and Astronomy, and Institute of Applied Physics, Seoul National University, Seoul 08826, Republic of Korea; Department of Biological Sciences, and KAIST Stem Cell Center, Korea Advanced Institute of Science and Technology, Daejeon 34141, Republic of Korea; Department of Physics and Astronomy, and Institute of Applied Physics, Seoul National University, Seoul 08826, Republic of Korea

## Abstract

Bacterial and bacteriophage RNA polymerases (RNAPs) have divergently evolved and share the RNA hairpin-dependent intrinsic termination of transcription. Here, we examined phage T7, T3 and SP6 RNAP terminations utilizing the single-molecule fluorescence assays we had developed for bacterial terminations. We discovered the phage termination mode or outcome is virtually single with decomposing termination. Therein, RNAP is displaced forward along DNA and departs both RNA and DNA for one-step decomposition, three-dimensional diffusion and reinitiation at any promoter. This phage displacement-mediated decomposing termination is much slower than readthrough and appears homologous with the bacterial one. However, the phage sole mode of termination contrasts with the bacterial dual mode, where both decomposing and recycling terminations occur compatibly at any single hairpin- or Rho-dependent terminator. In the bacterial recycling termination, RNA is sheared from RNA·DNA hybrid, and RNAP remains bound to DNA for one-dimensional diffusion, which enables facilitated recycling for reinitiation at the nearest promoter located downstream or upstream in the sense or antisense orientation. Aligning with proximity of most terminators to adjacent promoters in bacterial genomes, the shearing-mediated recycling termination could be bacterial adaptation for the facilitated reinitiations repeated at a promoter for accelerated expression and coupled at adjoining promoters for coordinated regulation.

## Graphical Abstract

**Graphical Abstract:**
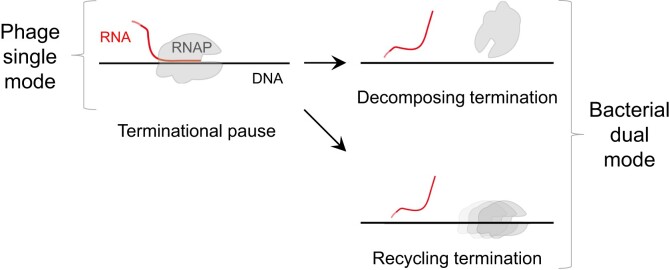

## Introduction

DNA-directed RNA polymerases (RNAPs) of bacteria and bacteriophages T7, T3, SP6 and others are regarded homologous with each other. They have markedly different subunit compositions with five bacterial subunits versus a single phage subunit but retain structurally conserved functional domains (1). The phage transcription terminations all occur intrinsically with RNAPs alone, while the bacterial terminations happen intrinsically without any auxiliary factors or extrinsically requiring the general transcription termination factor Rho (ρ).

The phage intrinsic termination can occur at two different classes of terminators, whereas the bacterial intrinsic termination only at class I but not class II. The class I terminators encode an RNA hairpin followed by a U-rich sequence (2,3). The class II terminators consist of a conserved sequence HATCTGTT (H = A, C or T) and a T-rich sequence (4–6). Thus, the phage-encoded and bacterial RNAPs perform the class I termination in common but have diversified into the class II termination or the ρ-dependent termination, respectively.

The phage U-rich sequence pauses RNAP at the termination site, providing time for the RNA hairpin to form and unwind the RNA·DNA hybrid at an upstream part, and then the remaining downstream part comprising mostly rU·dA base pairs becomes thermodynamically unstable, facilitating the RNA release (7). Thus, the phage and bacterial class I terminations are alike in the terminator-encoded structures and functions, although the details differ in the terminator oligo(U) length, hairpin stability, hybrid length and bubble size (2,8,9).

The bacterial termination modes or outcomes are twofold at any single terminator (10) as we and others have revealed with hairpin-dependent (11–16) and ρ-dependent terminators (17,18). First, in the decomposing termination, RNAP releases both RNA and DNA for decomposition, permitting three-dimensional diffusion for reinitiation at any promoter. Second, in the recycling termination, RNAP releases only RNA for one-dimensional (1D) diffusion and expeditious recycling on DNA, facilitating reinitiation at a nearby promoter.

Recently with the bacterial ρ-dependent terminators, we found that the decomposing termination is accomplished by a hypertranslocation-like displacing mechanism where RNAP is pushed or displaced forward on DNA, and the recycling termination by a shearing mechanism where RNA is pulled or sheared out of the RNA·DNA hybrid (17). Then, a question is whether the primitive RNAPs of such lytic phages perform the displacing-decomposing, the shearing-recycling or both mechanisms for their transcription termination.

In this study, we investigated the class I and II terminations of the phage T7, T3 and SP6 RNAP transcriptions, using the single-molecule assays that we had developed to explore the bacterial intrinsic and extrinsic terminations. The phage intrinsic terminations usually make only the decomposing outcome unlike the bacterial dual mode terminations. This suggests that the recycling termination is a bacterial gain and adaptation. It aligns with the observation that most terminators are located close to the nearest promotors in the bacterial genomes.

## Materials and methods

### Stalled transcription complexes

Transcription reactions were carried out with the phage T7, T3 and SP6 RNAPs separately in different buffers. They consist of 20 mM Tris–HCl, pH 8.0, 20 mM MgCl_2_, 20 mM NaCl and 1 mM dithiothreitol for T7 RNAP; 40 mM Tris–HCl, pH 7.9, 6 mM MgCl_2_, 10 mM NaCl, 2 mM spermidine and 10 mM dithiothreitol for T3 RNAP; and 40 mM Tris–HCl, pH 7.9, 6 mM MgCl_2_, 2 mM spermidine and 10 mM dithiothreitol for SP6 RNAP. These phage RNAPs are sensitive to proteolysis (4–6) but those purchased from Enzynomics, Korea were all intact.

For the transcription stalling, 15 units/μl RNAP and 10 nM biotinylated DNA labeled with Cy5 were incubated only with 150 μM ATP, 150 μM CTP and 250 μM Cy3-UTP (19) in a transcription buffer at 37°C for 20 min. The incubation was put into a microscope reaction chamber assembled with the quartz slide and cover slip that were coated with a 1:40 blend of biotinylated and unlabeled polyethylene glycol. Then, the stalled complexes were dispersedly fixed on their surfaces via biotin-streptavidin-biotin conjugation (20).

### Single-molecule transcription assays

The transcription buffer at 37°C in the chamber was supplemented with 5 mM protocatechuate acid and 100 nM protocatechuate-3,4-dioxygenase to mitigate the dye photobleaching (21) and additionally with saturated Trolox to minimize the blinking (22). After the immobilized stalled complexes were extensively washed to remove all the unbound, the transcription elongation was resumed by addition of all four NTPs to the reaction chamber using a syringe pump Fusion 100-X (Chemyx, USA).

The real-time fluorescent images were captured from the reaction samples on the quartz slide surface rather than the cover slip surface using a custom-made prism-type total internal reflection fluorescence microscope. The time resolution was 0.4 s with 0.2-s exposures in the alternating laser excitation mode (23). The Cy3 and Cy5 fluorophores were excited alternately using a 532-nm green laser Excelsior-532-50-CDRH and a 640-nm red laser Excelsior-640c-60-CDRH (Spectra-Physics, USA), respectively.

The single-molecule fluorescence signals were all collected with an UPlanSApo 60× lens (Olympus, Japan), filtered by NF03-532 E-25 and NF03-633 E-25 notch filters (Semrock, USA), separated with a 635dcxr dichroic mirror (Chroma, USA) and finally recorded by an iXon DU-897 camera (Andor, UK). Each set of data was collected from three independent experiments and analyzed using software IDL 7.0 (ITT, USA), MATLAB R2018a (MathWorks, USA), Excel 2016 (Microsoft, USA) or Origin 8.5 (OriginLab, USA).

### Bulk transcription assays

T7, T3 or SP6 RNAP of 15 units/μl, 10 nM template DNA and 200 μM NTPs were incubated together in the respective transcription buffer described above at 37°C for 30 min. The incubation was continued with 1.7 units/μl DNase I, RNase-free (Enzynomics, Korea) for 30 min and stopped with an equal volume of 178 mM Tris-borate, pH 8.0, 1.6 M urea and 4 mM ethylenediaminetetraacetic acid. The RNA products were separated by size on a 9% (w/v) polyacrylamide gel in 8 M urea and stained with GelRed^®^ (Biotium, USA).

### Template DNA preparation

The nontemplate strands of all the transcription templates were each prepared by ligation of a downstream 5′-phosphorylated donor oligomer and an upstream acceptor oligomer using a splint oligomer. The three respective oligomers were mixed at a molar ratio of 2:1:5 in an annealing buffer of 50 mM Tris–HCl, pH 8.0, 1 mM ATP, 10 mM MgCl_2_ and 10 mM dithiothreitol. After heated to 95°C, the mixture was slowly cooled down to 25°C by lowering 0.5°C every 30 s for annealing of the donor and the acceptor to the splint.

Then, T4 DNA Ligase (New England Biolabs, USA) was added to 40 units/μl in the annealing mixture for overnight incubation at 4°C. Each ligation product was amplified into a duplex DNA by a polymerase chain reaction with a biotin-labeled forward primer and a Cy5-labeled backward primer using the AccuPower Pfu PCR PreMix & Master Mix (Bioneer, Korea). All oligomers were custom-synthesized by Integrated DNA Technologies, USA or Bionics, Korea. Their sequences are listed in Supplementary Table S1.

For the mismatch template experiments, first, the wildtype DNAs were each produced with a biotin-labeled forward primer and an unlabeled backward primer, and the mutant DNAs each with unlabeled forward and Cy5-labeled backward primers (Supplementary Table S1). Then, the biotin-labeled and Cy5-labeled strands were mixed in the above annealing buffer, heated to 95°C and then cooled down to 4°C with stepwise decreases of 0.5°C per 30 s, allowing them to anneal together to form the duplex templates.

### Terminator–promoter distances

The genomic positions of transcription start sites of promoters and termination sites of terminators were retrieved from the RegulonDB database of *Escherichia coli* K-12 transcriptional regulatory network (24) and such a database of *Bacillus subtilis* (25). Using a home-made code, measured was the linear distance in base pairs between each termination site and the closest start site. The 1D distance distributions were then analyzed using Visual Studio 2017 (Microsoft, USA).

## Results

### Fluorescent transcription elongation complexes

In this study, we adapted the single-molecule fluorescence assays that we had earlier developed with *E. coli* RNAP and assorted bacterial terminators (11,13,17,18) to the phage T7, T3 and SP6 RNAP transcriptions (26–28). A major modification was that 5′-Cy3-ApU dinucleotide was incorporated at the first two residues of nascent RNAs by an *E. coli* RNAP holoenzyme, but Cy3-UTP mononucleotide was placed at an optimal position other than the 5′ end by the phage-encoded RNAPs (Supplementary Figure S1).

**Figure 1.**
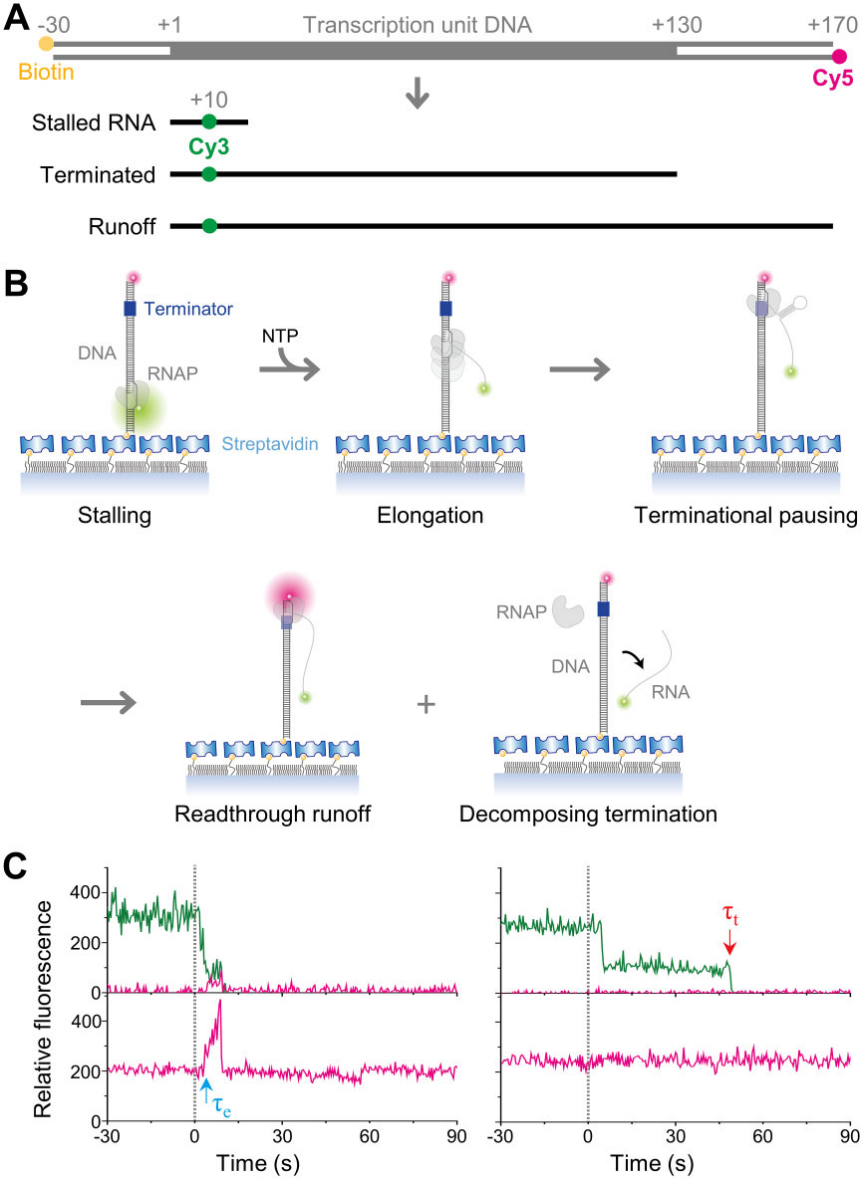
Transcription termination and readthrough monitored in the single-molecule assays. (**A**) Fluorescent labeling of template DNA and transcript RNA. Templates biotinylated at the upstream 5′ end were labeled with Cy5 at the downstream 5′ end. All transcripts were labeled with Cy3 at the 10th residue. Termination occurred mostly at the +130 position relative to the transcription start site +1. (**B**) Single-molecule assay scheme. The elongation complexes stalled with a 20-nt RNA were immobilized and exhibited Cy3 PIFE. When NTPs were injected at the time zero, RNAP resumed the elongation and moved away from the Cy3 so the Cy3 PIFE diminished in the active complexes. The transcription outcomes were only twofold, readthrough and termination. (**C**) Two distinct patterns of fluorescence changes. Shown are the fluorescence time traces of Cy3 (green) and Cy5 (red) at the Cy3 excitation (top) and the Cy5 excitation (bottom). When transcription readthrough occurred at the terminator being ignored (left traces), the continuously transcribing RNAP reached the Cy5-end at the time τ_e_ to start Cy5 PIFE. Then, the Cy5 PIFE ended with Cy3 signal vanishing, indicating that RNAP ran off the DNA end along with an extended Cy3-RNA. This is called readthrough runoff. Additionally, when transcription termination happened at the terminator being recognized (right traces), a Cy3-RNA transcript was released and diffused away at the time τ_t_. The absence of Cy5 PIFE indicates that RNAP also fell off DNA without reaching the Cy5-end. These transcription complexes decomposed into their RNA, DNA and RNAP components at once, so this mode is called decomposing termination.

The first linear DNA template in a series consisted of a 130-bp T7 RNAP transcription unit, a 30-bp upstream part and a 40-bp downstream part with blunt ends (Figure 1A). The downstream 5′ end was labeled with Cy5 for real-time monitoring, while the upstream 5′ end was biotinylated for individual immobilization. This 200-bp DNA sequence was designed first by fusion of two T7 genomic sequences of 50 bp around the *φ10* promoter (29) and 150 bp across the *Tφ* terminator (30) of class I (Supplementary Table S1).

The fusion was then followed by some substitutions at the +7 to +21 positions relative to the transcription start site +1 such that the initial transcription round could be stalled with a 20-nt RNA harboring a single Cy3-UMP at the 10th residue. The elongation stalling was achieved by the incubation of RNAP and Cy5-DNA only with ATP, GTP and Cy3-UTP but without CTP or UTP in a transcription buffer at 37°C for 20 min. Thus, the elongation complexes were double fluorescent with Cy3 on RNA and Cy5 on DNA.

### Single-molecule monitoring of transcription

The stalled elongation complexes were fixed on the microscope slides and thoroughly washed to get rid of the unbound including leftover Cy3-UTP so that the RNA transcripts did not get labeled any further. In our single-molecule transcription termination assays (Figure 1B), the transcription stalling was rescued by addition of all four unlabeled NTPs to resume the elongation. The NTP injection was timed zero in the experiments where the fluorescence of Cy3-RNA and Cy5-DNA was monitored on individual complexes in real time.

The RNAP stalled at +20/+21 with a 20-nt RNA directly contacted the Cy3 fluorophore placed at the RNA 10th residue to restrict the dye's photoisomerization from fluorescent to nonfluorescent isoform, which thereby invoked protein-induced fluorescence enhancement (PIFE) (31–34). This Cy3 PIFE diminished when the transcription got resumed and the elongating RNAP moved down away from the stalling site to liberate the dye. The complexes exhibiting this change were deemed active in transcription and solely analyzed here.

### Transcription termination and readthrough

Only two patterns of fluorescence changes were observed from all the active complexes (Figure 1C). First, after the Cy3 PIFE diminished, Cy5 PIFE started and then ended along with the Cy3 signal vanishing on some of the complexes. This pattern indicates that after the elongation resumption, transcription readthrough takes place at the terminator being ignored, and the continuously transcribing RNAP reaches the Cy5-end to yield the Cy5 PIFE and then runs off the end along with the extended Cy3-RNA diffusing away.

Second, the Cy3 signal faded away without the Cy5 PIFE occurrence on the remaining complexes. This indicates that the Cy3-RNA is released at the terminator being recognized, and concurrently RNAP falls off DNA without coming to the Cy5-end. These complexes decompose into their RNA, DNA and RNAP components at once, and this mode is called decomposing termination. Accordingly, each and every active transcription complex undergoes either the readthrough runoff or the decomposing termination.

**Figure 2.**
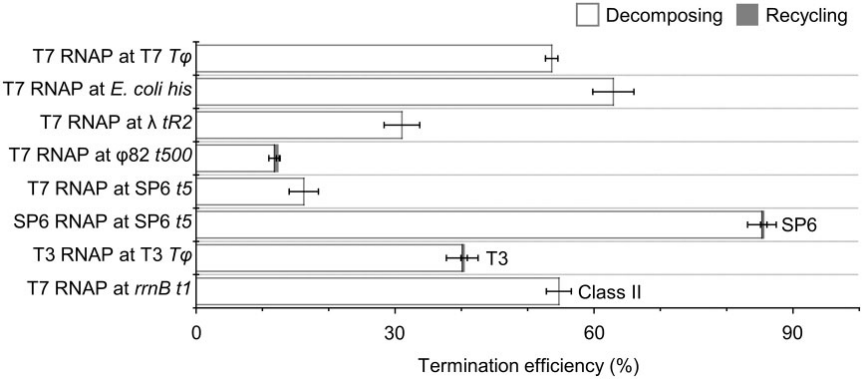
Virtual singleness of the phage transcription termination mode and the outcome. Examined were a total of eight pairs of T7, T3 or SP6 RNAP and class-I or -II terminator. The total termination efficiencies are shown as divided into the decomposing (blank) and recycling (filled) termination efficiencies. Note that the recycling termination is negligible, 0.1% overall. Error bar represents standard deviation of three independent datasets. The termination efficiency data are shown in Supplementary Table S2.

Such readthrough and termination events were identified at 5 min since the resumption. The event counting yielded the termination efficiency of 54 ± 1% (mean ± SD, Figure 2 and Supplementary Table S2), similar to the measurement in a bulk transcription assay, which additionally confirmed the single major termination site of +130 (Supplementary Figure S2). Photobleaching of Cy3 and Cy5 took 23 and 39 min (11), respectively (Supplementary Figure S3) so could hardly falsify the event classification at 5 min.

### Single-mode termination of phage RNAPs

The T7 termination leading only to the decomposing outcome contrasts sharply with the bacterial terminations. *E. coli* RNAP performs not only decomposing but also recycling terminations compatibly at any single bacterial terminator (10). The recycling termination of RNA-only release leaves RNAP on DNA for its 1D diffusion towards both ends of DNA for facilitated recycling (11–13). None of the T7 complexes (*n* = 1397) showed the recycling termination pattern that the Cy5 PIFE appears as RNAP translocates down to the Cy5-end after, not before, the Cy3 signal vanishes at the terminational release of Cy3-RNA.

Then, we examined T3 and SP6 RNAPs. Similar to the above T7 template, a T3 template was constructed first by fusion of two T3 genomic sequences of 50 bp around the *φ10* promoter and 150 bp across the *Tφ* terminator of class I, and an SP6 template with 50 bp of the *P8* promoter and 150 bp of the *t5* terminator of class I. These were modified in the sequence to permit Cy3-UMP labeling at the 10th residue and CTP-limited stalling with a 20-nt RNA. Only one (0.2%) of the T3 complexes (*n* = 474) and one (0.3%) of the SP6 complexes (*n* = 351) showed the recycling termination pattern (Figure 2).

Next, we tested five other terminators with T7 RNAP. They were *E. coli his* (*n* = 258), phage λ *tR2* (*n* = 390), phage φ82 *t500* (*n* = 672) and phage SP6 *t5* (*n* = 689) of class I, and *E. coli rrnB t1* (*n* = 289) of class II. The recycling termination was detected only on three complexes of *t500* (0.4%) among all those with the five terminators (Figure 2). Thus, its overall chance altogether in the eight cases was 0.1% (= 5/4520). Accordingly, the decomposing termination is almost a sole mode at any phage terminator of class I or II.

The decomposing termination efficiency ranged from 12% with T7 RNAP at *t500* to 85% with SP6 RNAP at *t5*, while the total termination efficiency from 12 to 86%, respectively (Figure 2 and Supplementary Table S2). These ranges are so broad to suggest that the virtual singleness of the phage termination mode is independent of the termination efficiencies. In the bulk assay, the termination site appeared nearly single with T7 RNAP at T7 *Tφ* and SP6 RNAP at SP6 *t5* but multiple with T3 RNAP at T3 *Tφ* (Supplementary Figure S2).

### Terminator-promoter distances in genomes

**Figure 3.**
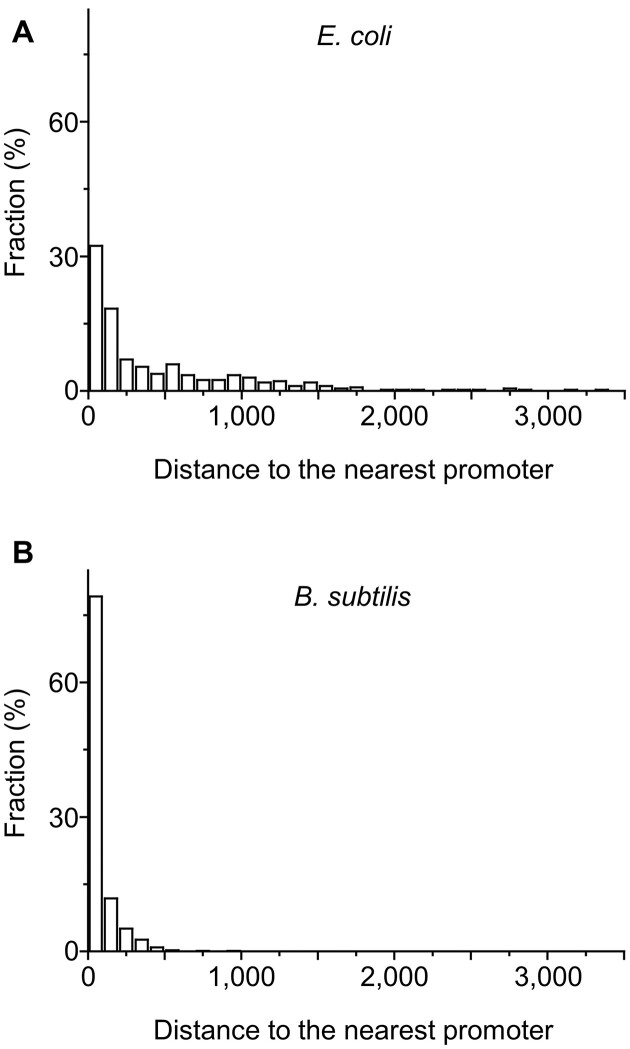
Bacterial genomic distances between the terminators and their nearest promoters. The linear distances in base pairs between the major termination sites and the start sites closest to them were measured in the *E. coli* (**A**) and *B. subtilis* (**B**) genomes. Their genomic positions were retrieved from the databases described in the Materials and methods section. The histograms represent the distributions of the distances with the bin width of 100 bp.

*E. coli* RNAP has been estimated to be able to reinitiate at a promoter after translocating on DNA up to about 1000 bp during the recycling stage after the recycling termination at physiological concentrations of the σ^70^ (RpoD) initiation factor (35). Based on this analysis, in the Gram-negative *E. coli* K-12 genome (24), 85% of the major termination sites (*n* = 371) are 1000 bp or less away from the nearest start sites, where the reinitiations thereby can be effectively facilitated (Figure 3A).

Likewise, in the Gram-positive *B. subtilis* genome (25), all the major termination sites (*n* = 888) are 952 bp or less away from their nearest start sites (Figure 3B). It is not known yet, however, how far *B. subtilis* RNAP can move on DNA during the recycling stage, if not similar to *E. coli* RNAP. Still, the recycling termination occurrence could give both Gram-positive and -negative bacteria definite advantages of the facilitated and coupled transcription reinitiations at the adjacent promoters under coregulation.

There are one T7, one T3 and seven SP6 RNAP terminators in their respective phage genomes, and the closest start sites are 14 to 1742 bp away from their termination sites (36–39). However, these distances would not matter, because such lytic phages produce their own RNAPs in large excess over their cognate promoters and exercise little sophisticated control during their lytic infection. Thus, the recycling termination for the facilitated reinitiation would not much benefit the phage life cycles.

### Slow decomposing termination of phage RNAPs

**Figure 4.**
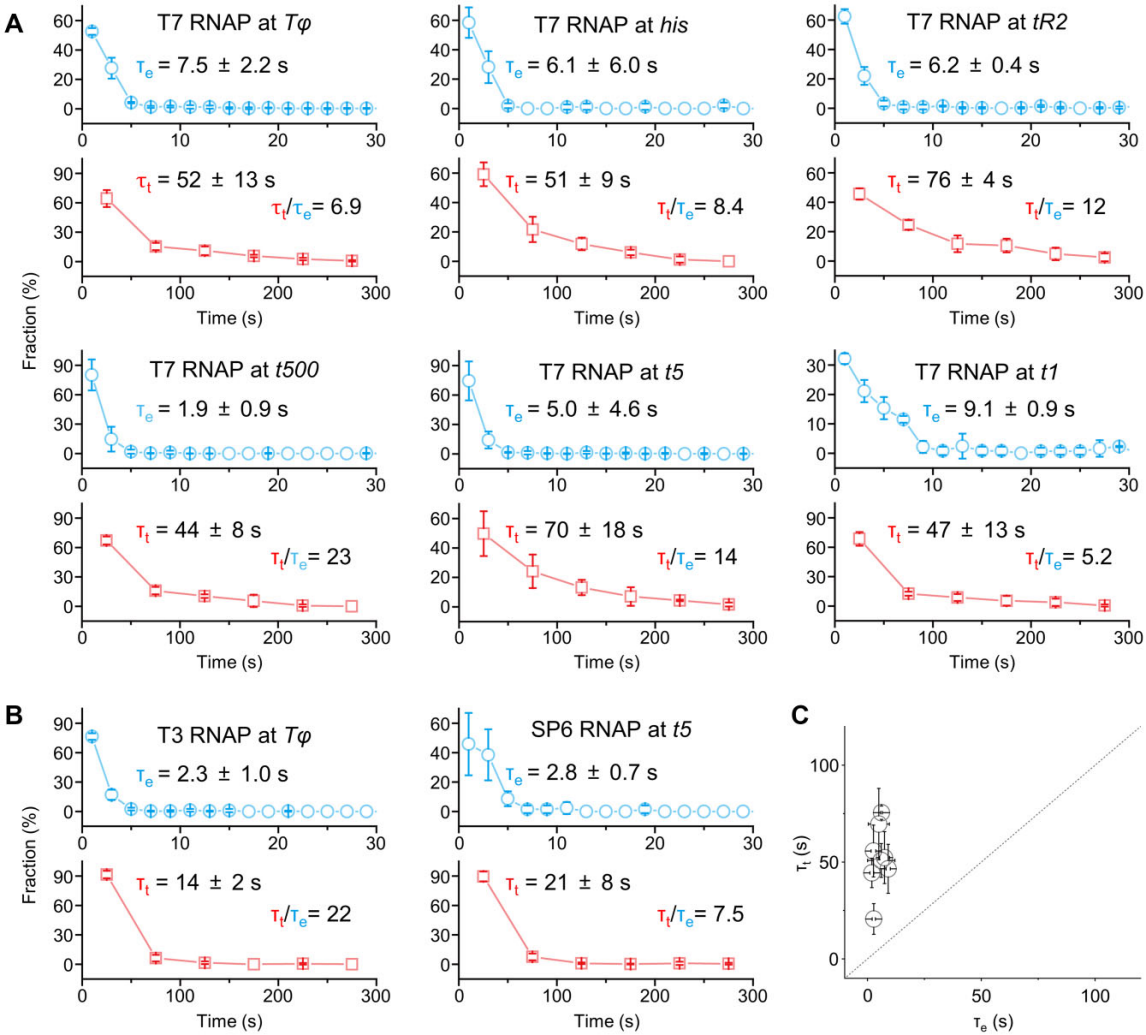
Time difference between the phage decomposing termination and the readthrough. (A, B) Distributions of the Cy5-end-reaching time τ_e_ and the termination time τ_t_. These times were measured and averaged in the assays using T7 RNAP with five class I terminators and one class II terminator (**A**) and additionally using T3 and SP6 RNAPs with their genomic terminators (**B**). Note that the x-scale is 10-fold larger for τ_t_ than τ_e_. (**C**) Direct comparisons of τ_e_ and τ_t_. In all eight RNAP-terminator pairs, τ_t_ was certainly later than τ_e_, which came after the readthrough occurred at the terminator. Error bar represents standard deviation of three independent datasets. The timing data are shown in Supplementary Table S3.

After the transcription readthrough at T7 *Tφ*, the continuously elongating T7 RNAP arrived at the Cy5-end to start the Cy5 PIFE at the time τ_e_ of 7.5 ± 2.2 s on average since the time zero of the NTP injection (Figure 4A and Supplementary Table S3). The readthrough time could not be directly measured but must be somewhat earlier than τ_e_. The termination occurred with the Cy3 signal vanishing at the time τ_t_ of 52 ± 13 s on average, 6.9-fold later than τ_e_ (Figure 4A). Thus, the decomposing termination is much delayed compared with the readthrough.

We then analyzed τ_e_ and τ_t_ on the complexes with the five other terminators of classes I and II listed above and T7 RNAP (Figure 4A) and additionally on the above-mentioned T3 and SP6 complexes (Figure 4B). Surely, τ_t_ of 21 to 76 s was later than τ_e_ of 1.9 to 9.1 s in all direct comparisons, where the fold delay τ_t_/τ_e_ ranged from 5.2 to 23 (Figure 4C). Thus, during the terminational pauses, the phage RNAPs go through a slow transition period in a long-lived inactivated state in order to complete the decomposing termination.

### RNAP displacement for decomposing termination

**Figure 5.**
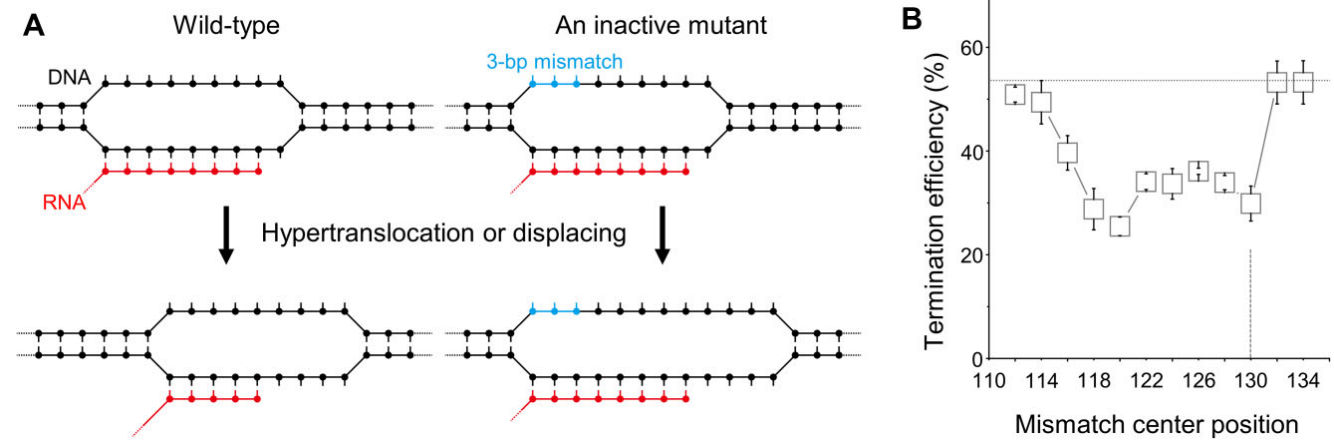
The RNAP displacing mechanism leading to the phage decomposing termination. (**A**) An experimental test for the hypertranslocation model of termination. RNAP and the transcription bubble both intrinsically translocate forward along DNA without any RNA extension, and consequently the RNA·DNA hybrid is shortened to facilitate the RNA release for termination according to the model. A 3-bp mismatch at the bubble's upstream edge would interfere with the DNA rewinding and the hybrid shortening to impair the decomposing termination more severely than the mismatches at any other positions. (**B**) Mutational effects of T7 *Tφ* terminator on the decomposing termination efficiency. The 3-bp mismatches centered at the twelve positions varying from +112, +114, +116 … to +134, while the major termination site was single at +130 (vertical line). Most mutants exhibited the decomposing termination efficiencies lower than the wildtype of 54% (horizontal line). Error bar represents standard deviation of three independent datasets. The termination efficiency data are shown in Supplementary Table S2.

We then examined whether the phage decomposing termination occurs through the RNAP displacing mechanism that we had previously demonstrated for the *E. coli* ρ-mediated decomposing terminations (17). We measured the termination efficiencies of T7 RNAP at a series of twelve T7 *Tφ* terminator mutants carrying a 3-bp mismatch at varying positions in a test of the hypertranslocation model (Figure 5) as previously described (17,40). The above-described practical singleness of the wildtype termination site lent validity to the test.

In the hypertranslocation model of termination, during the pause at the termination site, RNAP and the transcription bubble translocate forward on DNA without any RNA extension by hybrid unwinding and DNA rewinding at the bubble's upstream border, and the resulting hybrid shortening facilitates the RNA release (41,42). If so, a mismatch right at the upstream edge would interfere with the bubble rewinding, the hybrid shrinkage and the termination more severely than the mismatches at any other positions (Figure 5A).

Most mutations impaired the decomposing termination (Figure 5B), indicating that the bubble rewinding is critical for it. The effect peaked with the 3-bp mismatch centering at 10 bp upstream of the single major termination site +130. The peak is just outside of the 8-bp hybrid region in the T7 RNAP elongation complex structure (9). Thus, similar to our previous observation with *E. coli* RNAP terminations (17), the peak position is inconsistent with the hypertranslocation model, so the mechanism is called RNAP displacing instead.

### Trivial effects of excess RNAP on termination

**Figure 6.**
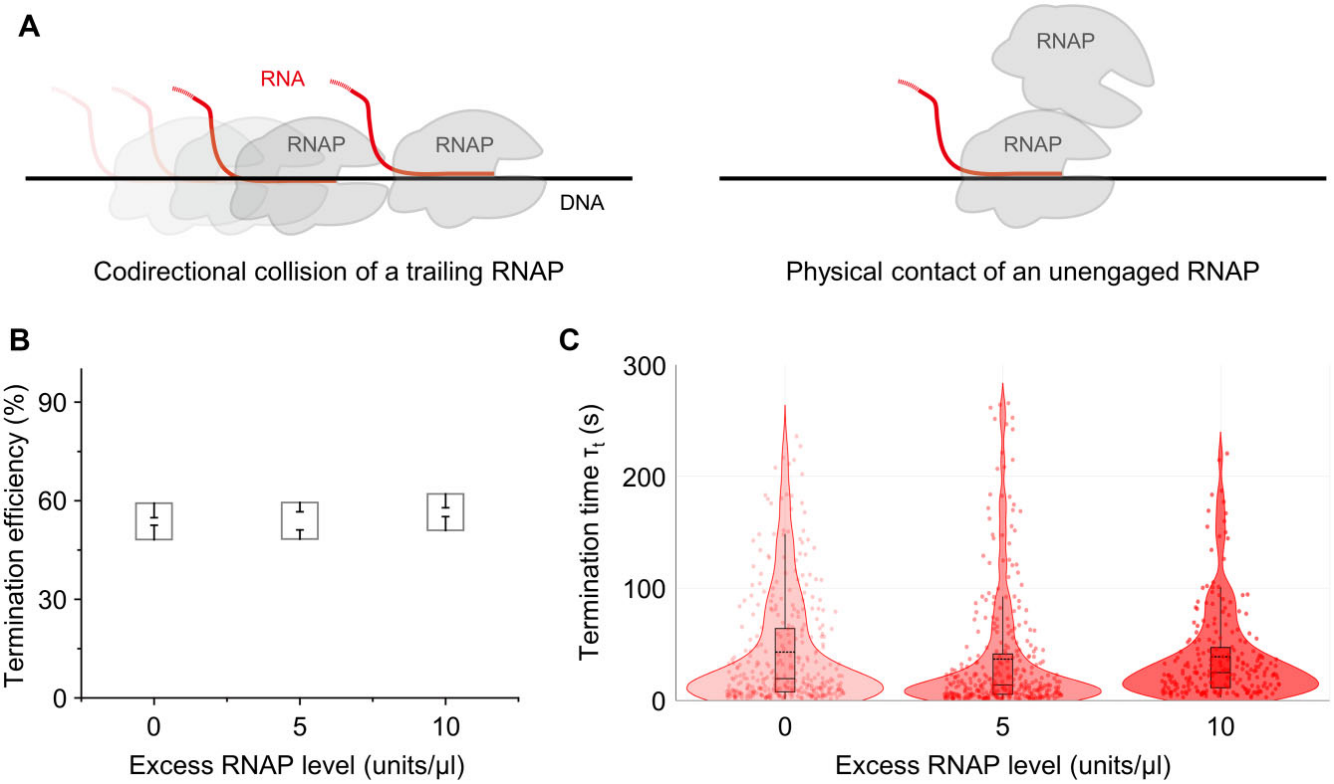
Trivial effects of the codirectional collision and the physical contact on termination. The unknown effects of extra T7 RNAP on T7 *Tφ* termination were examined by addition of RNAP in two levels to the resumption mixture in the single-molecule assays. (**A**) Two questioned interactions of surplus RNAP with the transcribing RNAP. One interaction (left) is that a trailing RNAP makes codirectional collision with the leading RNAP that is pausing most likely at the termination site because strong pause is absent at any other positions (Supplementary Figure S2). The other interaction (right) is that an unengaged RNAP comes into physical contact with the translocating or pausing RNAP at any DNA position in a random manner. (**B**) Termination efficiencies with excessive RNAP. For the RNAP supplement of 5 and 10 units/μl, a 50 units/μl stock of RNAP in the 50% glycerol-containing storage buffer (Enzynomics, Korea) was added to the resumption mixture in 10- and 5-fold dilutions, respectively. Hence, a negative control with 0 unit/μl RNAP had 5% glycerol. Error bar represents standard deviation of three independent datasets. The termination efficiency data are shown in Supplementary Table S2. (**C**) Distributions of the termination time τ_t_ with extra RNAP. Shown are the violin plots of the distributions without and with excess RNAP in the two levels. The timing data are shown in Supplementary Table S3.

The phage RNAPs are massively produced during the lytic cycle and amply used in the *in vitro* transcription reactions. So, we examined the effects of surplus RNAP on termination (Figure 6A). A question was whether termination can be facilitated by collision of RNAP at the termination site with a trailing RNAP, while without the recycling termination, the 1D diffusion of post-terminational RNAP is not available for such collision. Another question was whether premature termination can be caused by random contact with a free RNAP.

Here, we measured the effects of excessive T7 RNAP on the T7 *Tφ* termination. RNAP was added to the resumption NTP mixture, and the cases without and with the extra RNAP were compared in the termination efficiency and time τ_t_. With excess RNAP of 5 units/μl (and 5% glycerol, *n* = 617) and 10 units/μl (and 10% glycerol, *n* = 409), the termination mode was still only decomposing, and the efficiency was 54 ± 3% and 57 ± 1%, respectively similar to 54 ± 1% of a negative control without extra RNAP (with 5% glycerol, *n* = 642) (Figure 6B).

The termination time τ_t_ distributions were non-normal so compared using the Wilcoxon's rank sum test. The median was 14 and 25 s with additional RNAP, respectively compared with 19 s of the negative control, but the two-sided *P* value was 0.28, indicating that the differences were not significant (Figure 6C). Thus, the surplus RNAP little affects the termination efficiency and time. That is, the forward collision of a trailing RNAP or the physical contact of a free RNAP hardly enhances or hastens the termination.

## Discussion

The phage T7, T3 and SP6 RNAPs are the simplest consisting of a single subunit unlike the multisubunit RNAPs of bacteria, archaea and eukaryotes, and sensitive only to the lysozyme inhibition in contrast to the multiple positive and negative regulatory mechanisms in the organisms. Thus, the phage RNAPs would operate an elementary or basic mechanism in each stage of transcription. In this study, the phage mode of termination was discovered single, simpler than the bacterial dual mode.

The phage termination mechanism found here is that RNAP and the transcription bubble move forward on DNA intrinsically without RNA elongation during the terminational pause, similar to a hypertranslocation model (43) rather than a shearing model (42), both previously alluded to the T7 RNAP termination. Then, this RNAP displacement makes one-step decomposition of transcription complexes into their RNA, DNA and RNAP components. Presumably, such displacement collapses the RNA·DNA hybrid to loosen RNA and changes the RNAP conformation to lose both RNA and DNA.

This displacing-decomposing mechanism is experimentally evidenced in this study by the bubble rewinding/translocation impediment of certain DNA mismatches and by the coincident or near-coincident dissociation of RNA and RNAP from the immobilized DNA, respectively. Resulting from such one-step decomposition at the decomposing termination, RNAP would diffuse three-dimensionally and reinitiate transcription randomly at any promoter in an unfacilitated manner, entering a transcription cycle.

The phage mechanism requires the bubble rewinding for termination, similar to the *E. coli* class-I termination (15,40,44), but is not identical to the hypertranslocation model. The rewinding hampering effect should peak with a mismatch at the bubble's upstream edge according to the model. However, the peak is not within but right outside of the T7 bubble. With *E. coli* RNAP, we have found the same, i.e. the peak is just beyond the *E. coli* bubble (17). Thus, the displacing-decomposing mechanism appears homologous between the phages and bacteria.

In contrast to the phage RNAPs, *E. coli* and *Saccharomyces cerevisiae* RNAPs perform both decomposing and recycling terminations compatibly at a single terminator. This dual mode has been found at the bacterial hairpin-dependent (11–13), ρ-dependent (17,18) and yeast Sen1-dependent terminators (45). The helicase Sen1 is an ortholog of bacterial ρ and human senataxin SETX. Accordingly, the recycling termination, lacking from the phage transcriptions, is a bacterial gain and likely the adaptation conserved at least in yeast.

In the recycling termination, RNA is sheared out of the hybrid (17,18) and then RNAP translocates and recycles on DNA for reinitiation in a facilitated manner at nearby promoters located downstream or upstream in the sense or antisense orientation, usually at the promoter closest to the termination site. Such 1D or facilitated diffusion and following reinitiation have been demonstrated, as the recycling RNAP can move downward and upward on DNA with the hopping and sliding motions, switch the direction and even flip its orientation (11–13).

The facilitated reinitiation after the bacterial recycling termination has been measured fairly efficient *in vitro* (11–13). It can take place proficiently at the original promoter after RNAP’s backward movement to enable speedy and persistent recurrence of multiple transcription rounds in an operon or gene for expedited expression. It is additionally competent at other closely adjacent promoters to couple the transcriptions of two or more neighboring and overlapping operons and genes for coordinated regulation.

It has been estimated plausible in *E. coli* (35), although not demonstrated *in vivo* yet. Its efficiency primarily depends on the availability of active sigma initiation factors and the 1D diffusion distances rather than the diffusion directions or the promoter orientations (11,13,35). In both Gram-negative *E. coli* and Gram-positive *B. subtilis* genomes, the linear distances between terminators and their nearest promoters are mostly shorter than a limit of the facilitated diffusion (35) and likely in other bacterial genomes as well.

The genomic vicinity of coregulated operons/genes has been shown to contribute to high levels of their coexpression in *E. coli* and evolutionarily conserved in more than 250 species of *Gammaproteobacteria* class including *E. coli* (46). This proximity would favor the facilitated reinitiation after recycling termination over the unfacilitated reinitiation after decomposing termination. Thus, the recycling termination capability could give definitive advantages of gene coregulation and round repetition to bacteria but not necessarily the lytic phages.

The class I terminator-encoded RNA hairpins causing the phage and bacterial intrinsic terminations are very much alike in their structures. So, the phages and bacteria have long been conjectured to share the same or similar mechanisms of action for their hairpin-dependent intrinsic terminations. In this study, however, their termination modes and outcomes were uncovered to be markedly distinct with the sole versus dual modes that are almost exclusive to the phage and bacterial terminations, respectively.

In fact, *E. coli his*, λ *tR2* and φ82 *t500* had shown the dual mode with *E. coli* RNAP in our previous study where the decomposing portion of the two modes was 13%, 9% and 30%, respectively (11), but all exhibited the sole mode here with T7 RNAP. Moreover, at T7 *Tφ*, T7 RNAP mode was single but *E. coli* RNAP mode was double with 64% decomposing mode (data not shown) in this study. Thus, whether the mode is sole or dual and whether the recycling termination occurs or not depend on RNAPs rather than terminators.

The phage and bacterial RNAPs share several structurally conserved domains such as DNA entry, DNA exit, NTP entry and RNA exit channels around the Mg^2+^-binding active site. In fact, the bacterial class-I terminators are often effective for the phage RNAPs and vice versa (11,41), although their efficiencies greatly vary, even between T7 (16%) and SP6 (86%) RNAPs at *t5*. The T7 RNAP’s S43 residue, the *E. coli* RNAP β subunit's flap domain and others are involved in termination (15,47) but it remains to be identified what residue(s) or domain(s) prevent or permit the recycling termination.

Termination efficiency can be varied by the reaction conditions and any transcribed sequences including those beyond the terminator in *cis-* and *trans*-acting manners (48–50), so any *in vitro* measurement can differ from the real *in vivo* efficiency. However, this and previous studies examined the assorted terminators with widely varied efficiencies of 12–86% and 18–84% by the phage and *E. coli* RNAPs (11,13,17,18), respectively. Therefore, their sole and dual mode characters appear general regardless of the efficiency levels.

The ρ-free intrinsic termination at the bacterial ρ-dependent terminators leads only to the decomposing outcome, while it is slower than the other three ρ-mediated termination routes to serve a fail-safe mechanism (17,18). This ρ-free termination shares the same single outcome of decomposition with the phage terminations. Hence, implicatively in bacteria, the decomposing termination is the basis to that the recycling termination has been added by the hairpin mediation and the factor dependability by the ρ presence.

The decomposing termination appears more general or less specific than the recycling termination. The decomposing outcome can be diversely achieved either intrinsically without and with RNA hairpin formation or extrinsically with the ρ factor in the phages and bacteria, whereas the recycling outcome comes only with hairpins or ρ in bacteria (11–13,17,18). Moreover, the hairpin-dependent termination yields the recycling outcome more often than the ρ-dependent termination so is better suited to the facilitated reinitiation.

Kinetically, the phage decomposing terminations are all slower than the readthrough, and take 0.7–1.3 min depending on terminators and RNAPs. This range is slower than the bacterial hairpin-dependent recycling and decomposing terminations of 0.06–0.4 min, similar to the ρ-mediated recycling route of 0.4–2 min and faster than any ρ-mediated decomposing path of 3–9 min (10,11,17,18). Thus, as to the decomposing mode alone, the bacterial class I terminations are faster but the ρ-mediated ones are slower than the phage ones.

During the virulent infection, the phage RNAPs are massively produced and surplus RNAP may influence the termination in two potential ways. One is another transcribing RNAP makes codirectional collision with the leading RNAP in pause. The other is an unengaged RNAP makes random contact with the translocating or pausing RNAP. Both of them can arise also in the *in vitro* transcription reactions with excessive RNAP. Here, we simulated both situations together by adding RNAP to the resumption mixture.

We found that the collision or the contact barely enhances or hastens termination. This is similar to a recent finding on *E. coli* termination. The bidirectional terminators are located between two convergent genes (51). A model is that two facing RNAPs stick together at the terminator and get dissociated by the collision with another RNAP coming next, but a single RNAP at it fails to get the collision-induced termination (16,52). The latter resembles that the phage RNAPs are seldom paired at a terminator and little affected by extra RNAP.

The phage transcriptions are widely used for *in vitro* and *in vivo* orthogonal syntheses of RNA and the termination defines the 3′ end of synthetic RNAs such as RNA therapeutics (53–57). Usually the most wanted is 100% termination at a single site. Among those tested here, SP6 RNAP at *t5* was the best with an efficiency of 86% and a major site. Alternatively, a tandem array of multiple imperfect terminators can achieve near perfection in a fail-safe system (10) but with heterogenous 3′ ends (58).

Various single-molecule techniques have been employed to investigate the transcription termination mechanisms. They enable precise characterization of the transient intermediate states and the diverse termination pathways that are often obscured in the traditional ensemble assays. Additionally, they yield more dependable kinetics data. Single-molecule fluorescence measurements, exclusively used here, provide the highest throughput and allow for testing a variety of reaction conditions with relative ease (11–13,16–18).

Optical and magnetic tweezers can exert mechanical stresses such as tension and supercoiling, unveiling how transcription responds to these stimuli (41,59). While limited to the analysis of post-termination RNA products, the nanopore technique can offer quantitative data on alternative terminations (58). The integration of distinct measurement modalities in novel ways using the single-molecule techniques is anticipated to address many unresolved questions in the transcription termination (16).

In summary, we show that the phage T7, T3 and SP6 RNAPs all terminate transcription in a virtually sole mode of the RNAP displacing mechanism for the decomposing termination at their class I and II terminators. These phage decomposing terminations appear homologous with the bacterial ones, being slower or faster. These results suggest that the RNA shearing mechanism for the recycling termination is bacterial adaptation to their compact genomes for the facilitated reinitiations and the coupled transcriptions.

## Supplementary Material

gkae620_Supplemental_File

## Data Availability

All data are available from the corresponding authors upon reasonable request.

## Notes

Present address: Eunho Song, Laboratory of Chemical Physics, National Institute of Diabetes and Digestive and Kidney Diseases, National Institutes of Health, Bethesda, MD 20892, USA.

Present address: Heesoo Uhm, Department of Biochemistry and Molecular Biology, and Medical Research Center of Genomic Medicine Institute, Seoul National University College of Medicine, Seoul 03080, Republic of Korea.
