# Supplementary material for: Single-mode termination of phage transcriptions, disclosing bacterial adaptation for facilitated reinitiations
Source: Nucleic Acids Res. 2024 Jul 16;52(15):9092–102. doi: 10.1093/nar/gkae620 (PMC11347151; doi:10.1093/nar/gkae620)
Supplement: gkae620_Supplemental_File [file gkae620_supplemental_file.docx]

**Supplementary Information**

**Single-mode termination of phage transcriptions, disclosing bacterial adaptation for facilitated reinitiations**

Eunho Song^1,†^, Sun Han^1,†^, Heesoo Uhm^1^, Changwon Kang^2,^* and Sungchul Hohng^1,^*

^1^ Department of Physics and Astronomy, and Institute of Applied Physics, Seoul National University, Seoul 08826, Republic of Korea

^2^ Department of Biological Sciences, and KAIST Stem Cell Center, Korea Advanced Institute of Science and Technology, Daejeon 34141, Republic of Korea

* To whom correspondence should be addressed. Email: shohng@snu.ac.kr

Correspondence may also be addressed to Changwon Kang. Email: ckang@kaist.ac.kr

†The first two authors should be regarded as Joint First Authors.

Present addresses:

Eunho Song, Laboratory of Chemical Physics, National Institute of Diabetes and Digestive and Kidney Diseases, National Institutes of Health, Bethesda, MD 20892, USA.

Heesoo Uhm, Department of Biochemistry and Molecular Biology, and Medical Research Center of Genomic Medicine Institute, Seoul National University College of Medicine, Seoul 03080, Republic of Korea.

**Contents**

Supplementary Figure S1: Optimization of the Cy3-UTP incorporation position

Supplementary Figure S2: Bulk transcription assays of the phage RNAPs

Supplementary Figure S3: Photobleaching times

Supplementary Table S1: Oligonucleotide sequences

Supplementary Table S2: Termination efficiencies

Supplementary Table S3: End-reaching and termination times

**Supplementary Figures**

**
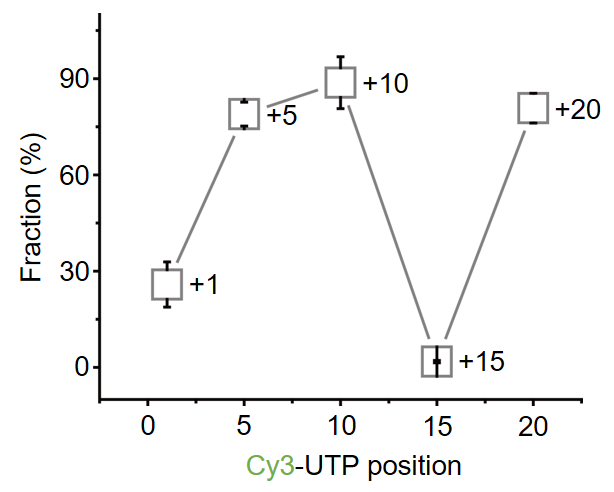
Supplementary Figure S1.** Optimization of the Cy3-UTP incorporation position. The fractions of DNA that form stalled T7 RNAP elongation complexes with a single Cy3-UMP-carrying RNA on the y-axis are plotted against the incorporation positions of the *Tφ* terminator template on the x-axis. The varying positions were the transcription start site +1, +5, +10, +15 and +20 positions. Among them, the 10th residue of RNA is the best and the 15th residue is the worst for the incorporation of Cy3-UMP. Error bar represents standard deviation of five independent datasets.

| Cy3-UMP position | Elongation complex formation % | *n* in replicated experiments |
| --- | --- | --- |
| +1 | 25.8 ± 7.0 | 3,187 = 658 + 657 + 629 + 660 + 584 |
| +5 | 78.9 ± 3.7 | 4,243 = 824 + 869 + 843 + 873 + 834 |
| +10 | 88.7 ± 8.1 | 3,196 = 461 + 737 + 706 + 677 + 615 |
| +15 | 1.8 ± 0.4 | 2,912 = 623 + 643 + 557 + 571 + 518 |
| +20 | 80.8 ± 4.6 | 2,789 = 587 + 524 + 602 + 511 + 574 |

**
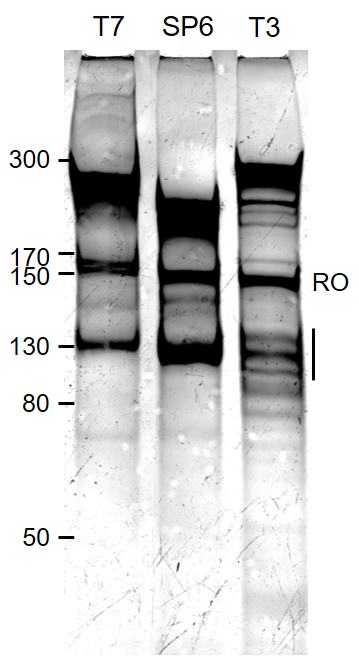
Supplementary Figure S2.** Bulk transcription assays of the phage RNAPs. The *in vitro* bulk transcription reactions using the phage T7 (left), SP6 (center) or T3 (right) RNA polymerase were carried out with the linear template harboring *Tφ* of the T7 genome, *t5* of the SP6 genome or *Tφ* of the T3 genome, respectively at 37°C for 30 min. The run-off transcripts are denoted by RO and the terminated transcripts by a vertical line.

**Supplementary Figure S3.** Photobleaching time. We measured photobleaching time of Cy3 with injection of an imaging buffer only (*n* = 471). The fractions of molecules with Cy3 fluorescence on the y-axis are plotted as a function of post-injection time on the x-axis, and fit to single exponential functions. The Cy3 photobleaching time was 1,355 s. The photobleaching time of Cy5, the other fluorophore used in this study, was previously reported as 2,350 s in *Nat. Commun*. (2020) 11, 450, where Cy5 was used under the same conditions.


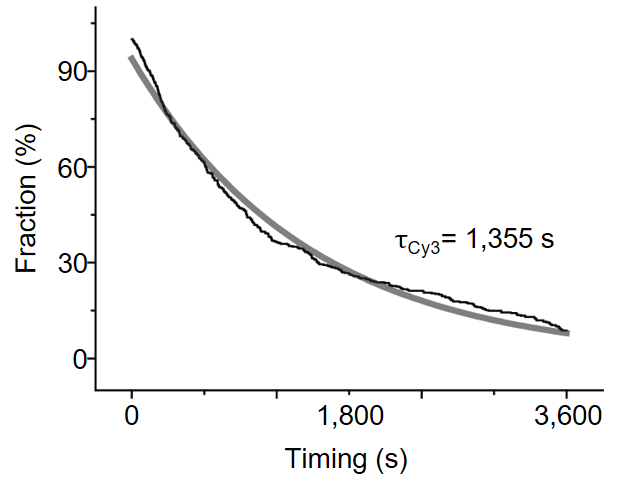


**Supplementary Tables**

**Supplementary Table S1.** Oligonucleotide sequences

Lowercase letters in red indicate mutations. p means a phosphate group.

1. Terminator templates of 200 bp for T7 RNA polymerase

Template was constructed by ligation of a T7-A fragment and a T7-B fragment with the T7-AB splint. The ligation products were amplified by polymerase chain reaction (PCR) using a common biotin-labeled forward primer 5'-TAGACTTCGAAATTAATACGACTCAC-3' and a respective Cy5-labeled backward primer, 5'-CGCCAAGCTTTCACCG-3' for *E. coli* *rrnB* *t1* terminator, or 5'-TACATCACTTGATTGTTGAATTACAGG-3' for SP6 *t5* terminator, or 5'-ACGTTCATATCGTATGAGCG-3' for the other terminators.

| Name | Length | | Sequence in the 5' to 3' direction |
| --- | --- | --- | --- |
| T7-A for Cy3-U at +1 | | 100 nt | TAGACTTCGAAATTAATACGACTCACTATAtGGAGAggAGAAgGGaaaggcCCGCGCTGCTAACAAAGCCCGAAAGGAAGCTGAGTTGGCTGCTGCCACC |
| T7-A for Cy3-U at +5 | | 100 nt | TAGACTTCGAAATTAATACGACTCACTATAGGGAtAggAGAAgGGaaaggcCCGCGCTGCTAACAAAGCCCGAAAGGAAGCTGAGTTGGCTGCTGCCACC |
| T7-A for Cy3-U at +10 | | 100 nt | TAGACTTCGAAATTAATACGACTCACTATAGGGAGAggAtAAgGGaaaggcCCGCGCTGCTAACAAAGCCCGAAAGGAAGCTGAGTTGGCTGCTGCCACC |
| T7-A for Cy3-U at +15 | | 100 nt | TAGACTTCGAAATTAATACGACTCACTATAGGGAGAggAGAAgGtaaaggcCCGCGCTGCTAACAAAGCCCGAAAGGAAGCTGAGTTGGCTGCTGCCACC |
| T7-A for Cy3-U at +20 | | 100 nt | TAGACTTCGAAATTAATACGACTCACTATAGGGAGAggAGAAgGGaaagtcCCGCGCTGCTAACAAAGCCCGAAAGGAAGCTGAGTTGGCTGCTGCCACC |
| T7-AB | | 40 nt | TATGCTAGTTATTACTCAGCGGTGGCAGCAGCCAACTCAG |
| T7-B for *T7 Tφ* | | 100 nt | pGCTGAGTAATAACTAGCATAACCCCTTGGGGCCTCTAAACGGGTCTTGAGGGGTTTTTTGCTGAAAGGAGGAACTATATGCGCTCATACGATATGAACGT |
| T7-B for *t1* | | 86 nt | pGCTGAGTAATAACTAGCATgggagaccacaacggtttccctctagaggatccgttttatctgttgtttgtcggtgaaagcttggcg |
| T7-B for *his* | | 97 nt | pGCTGAGTAATAACTAGCATgcccccggaagatgcatcttccgggggcttttttttttCTGAAAGGAGGAACTATATGCGCTCATACGATATGAACGT |
| T7-B for *tR2* | | 90 nt | pGCTGAGTAATAACTAGCATggcctgctggtaatcgcaggcctttttatttCTGAAAGGAGGAACTATATGCGCTCATACGATATGAACGT |
| T7-B for *t*500 | | 86 nt | pGCTGAGTAATAACTAGCATgcccgccgaaaggcgggcttttctgtCTGAAAGGAGGAACTATATGCGCTCATACGATATGAACGT |
| T7-B for *t5* | | 95 nt | pGCTGAGTAATAACTAGCATccctatctaccttgcgtaggtagggttcttttgtttaggaggattcatgcctgtaattcaacaatcaagtgatgta |
| T7-B for mismatch at +112 | | 100 nt | pGCTGAGTAATAACTAGCATAACCCCTTGGGGCCTCTAAACaaaTCTTGAGGGGTTTTTTGCTGAAAGGAGGAACTATATGCGCTCATACGATATGAACGT |
| T7-B for mismatch at +114 | | 100 nt | pGCTGAGTAATAACTAGCATAACCCCTTGGGGCCTCTAAACGGcagTTGAGGGGTTTTTTGCTGAAAGGAGGAACTATATGCGCTCATACGATATGAACGT |
| T7-B for mismatch at +116 | | 100 nt | pGCTGAGTAATAACTAGCATAACCCCTTGGGGCCTCTAAACGGGTgaaGAGGGGTTTTTTGCTGAAAGGAGGAACTATATGCGCTCATACGATATGAACGT |
| T7-B for mismatch at +118 | | 100 nt | pGCTGAGTAATAACTAGCATAACCCCTTGGGGCCTCTAAACGGGTCTactGGGGTTTTTTGCTGAAAGGAGGAACTATATGCGCTCATACGATATGAACGT |
| T7-B for mismatch at +120 | | 100 nt | pGCTGAGTAATAACTAGCATAACCCCTTGGGGCCTCTAAACGGGTCTTGtccGGTTTTTTGCTGAAAGGAGGAACTATATGCGCTCATACGATATGAACGT |
| T7-B for mismatch at +122 | | 100 nt | pGCTGAGTAATAACTAGCATAACCCCTTGGGGCCTCTAAACGGGTCTTGAGaaaTTTTTTGCTGAAAGGAGGAACTATATGCGCTCATACGATATGAACGT |
| T7-B for mismatch at +124 | | 100 nt | pGCTGAGTAATAACTAGCATAACCCCTTGGGGCCTCTAAACGGGTCTTGAGGGcaaTTTTGCTGAAAGGAGGAACTATATGCGCTCATACGATATGAACGT |
| T7-B for mismatch at +126 | | 100 nt | pGCTGAGTAATAACTAGCATAACCCCTTGGGGCCTCTAAACGGGTCTTGAGGGGTaaaTTGCTGAAAGGAGGAACTATATGCGCTCATACGATATGAACGT |
| T7-B for mismatch at +128 | | 100 nt | pGCTGAGTAATAACTAGCATAACCCCTTGGGGCCTCTAAACGGGTCTTGAGGGGTTTaaaGCTGAAAGGAGGAACTATATGCGCTCATACGATATGAACGT |
| T7-B for mismatch at +130 | | 100 nt | pGCTGAGTAATAACTAGCATAACCCCTTGGGGCCTCTAAACGGGTCTTGAGGGGTTTTTacgTGAAAGGAGGAACTATATGCGCTCATACGATATGAACGT |
| T7-B for mismatch at +132 | | 100 nt | pGCTGAGTAATAACTAGCATAACCCCTTGGGGCCTCTAAACGGGTCTTGAGGGGTTTTTTGgacAAAGGAGGAACTATATGCGCTCATACGATATGAACGT |
| T7-B for mismatch at +134 | | 100 nt | pGCTGAGTAATAACTAGCATAACCCCTTGGGGCCTCTAAACGGGTCTTGAGGGGTTTTTTGCTcttAGGAGGAACTATATGCGCTCATACGATATGAACGT |
| 2. Terminator template of 200 bp for SP6 RNA polymerase  The template was constructed by ligation of the SP6-A and SP6-B fragments with the SP6-AB splint. The ligation products were amplified by PCR using a biotin-labeled forward primer 5'-TCTTTAATTGCCTATTTAGGTGACAC-3' and a Cy5-labeled backward primer, 5'-TACATCACTTGATTGTTGAATTACAGG-3'. | | | |
| Name | | Length | Sequence in the 5' to 3' direction |
| SP6-A | | 100 nt | TCTTTAATTGCCTATTTAGGTGACACTATAGAAGGGAGGtAGaggagggacTGCTGCTGTTCGTGCTGTGATGGCTAATGACATCAAGCCGACTGCACTG |
| SP6-AB | | 40 nt | TTTGCATATTAGTAGATTGTTATCCCAATCACAACGAGCT |
| SP6-B | | 100 nt | AAGCCGACCGAGGAATAACCTATGCCCTATCTACCTTGCGTAGGTAGGGTTCTTTTGTTTAGGAGGATTCATGCCTGTAATTCAACAATCAAGTGATGTA |
|  | | | |
| 3. Terminator template of 200 bp for T3 RNA polymerase  The template was constructed by ligation of the T3-A and T3-B fragments with the T3-AB splint. The ligation products were amplified by PCR using a biotin-labeled forward primer 5'-TCTTTAATTGCCTATTTAGGTGACAC-3' and a Cy5-labeled backward primer, 5'-TACATCACTTGATTGTTGAATTACAGG-3'. | | | |
| Name | | Length | Sequence in the 5' to 3' direction |
| T3-A | | 100 nt | GACTCCAGTTTCTAATTAACCCTCACTAAAGGGAGAGAgtAaAGAaGggacACGACTATCATGTGGGCAACCACCCTGAAAGCTCGTTGTGATTGGGATA |
| T3-AB | | 40 nt | GGTTATTCCTCGGTCGGCTTCAGTGCAGTCGGCTTGATGT |
| T3-B | | 100 nt | ACAATCTACTAATATGCAAACCCCTTGGGTTCCCTCTTTGGGAGTCTGAGGGGTTTTTTGCTTTAACCCTCACTAACAGGAGGTAACATCATGCGCTCTT |

**Supplementary Table S2.** Termination efficiencies

The standard deviation is calculated from three independent datasets.

| 1. Termination efficiencies of phage RNAPs at wildtype terminators | | | | | | |
| --- | --- | --- | --- | --- | --- | --- |
| NAP at terminator | Decomposing termination (%) | | | Recycling termination (%) | | *n* in replicated experiments |
| T7 RNAP at T7 *Tφ* | 53.6 ± 0.9 | | | 0.0 ± 0.0 | | 1,397 = 447 + 569 + 381 |
| T7 RNAP at *t1* | 54.7 ± 1.9 | | | 0.0 ± 0.0 | | 289 = 105 + 89 + 95 |
| T7 RNAP at *tR2* | 31.0 ± 2.7 | | | 0.0 ± 0.0 | | 390 = 152 + 127 + 111 |
| T7 RNAP at *his* | 62.9 ± 3.1 | | | 0.0 ± 0.0 | | 258 = 77 + 91 + 90 |
| T7 RNAP at *t500* | 11.8 ± 0.8 | | | 0.5 ± 0.2 | | 672 = 137 + 354 + 181 |
| T7 RNAP at *t5* | 16.2 ± 2.2 | | | 0.0 ± 0.0 | | 689 = 229 + 278 + 182 |
| T3 RNAP at T3 *Tφ* | 39.9 ± 2.6 | | | 0.3 ± 0.5 | | 474 = 182 + 172 + 120 |
| SP6 RNAP at *t5* | 85.3 ± 2.2 | | | 0.3 ± 0.5 | | 351 = 144 + 85 + 122 |
| 2. Termination efficiencies of T7 RNAP at *Tφ* mismatch mutants | | | | | | |
| *Tφ* mutants | Decomposing termination (%) | | Recycling termination (%) | | | *n* in replicated experiments |
| mismatch at +112 | 50.9 ± 1.4 | | 0.3 ± 0.4 | | | 295 = 97 + 65 + 133 |
| mismatch at +114 | 49.4 ± 4.2 | | 0.7 ± 1.3 | | | 161 = 48 + 46 + 67 |
| mismatch at +116 | 39.6 ± 3.3 | | 0.0 ± 0.0 | | | 171 = 68 + 67 + 36 |
| mismatch at +118 | 28.8 ± 4.0 | | 0.0 ± 0.0 | | | 209 = 59 + 54 +96 |
| mismatch at +120 | 25.5 ± 1.8 | | 0.0 ± 0.0 | | | 258 = 51 + 152 + 55 |
| mismatch at +122 | 34.1 ± 1.5 | | 0.0 ± 0.0 | | | 200 = 71 + 78 + 51 |
| mismatch at +124 | 33.7 ± 2.9 | | 0.8 ± 0.7 | | | 250 = 92 + 81 + 77 |
| mismatch at +126 | 36.1 ± 0.6 | | 0.7 ± 1.2 | | | 108 = 47 + 31 + 30 |
| mismatch at +128 | 33.9 ± 1.4 | | 0.5 ± 0.9 | | | 216 = 62 + 72 + 82 |
| mismatch at +130 | 29.9 ± 3.4 | | 1.0 ± 1.1 | | | 313 = 138 + 94 + 81 |
| mismatch at +132 | 53.2 ± 4.1 | | 0.0 ± 0.0 | | | 150 = 56 + 56 + 38 |
| mismatch at +134 | 53.2 ± 4.2 | | 0.0 ± 0.0 | | | 157 = 60 + 33 + 64 |
| 3. Termination efficiencies of T7 RNAP with RNAP supplements | | | | | | |
| RNAP supplement | | Decomposing termination (%) | | | Recycling termination (%) | *n* in replicated experiments |
| 0 units/μl, 5% glycerol | | 53.7 ± 1.2 | | | 0.0 ± 0.0 | 642 = 186 + 216 + 240 |
| 5 units/μl, 5% glycerol | | 53.9 ± 2.8 | | | 0.0 ± 0.0 | 617 = 241 + 234 + 142 |
| 10 units/μl, 10% glycerol | | 56.5 ± 1.3 | | | 0.0 ± 0.0 | 409 = 138 + 148 + 123 |

**Supplementary Table S3.** End-reaching (τ_e_) and termination (τ_t_) times
The standard deviation is calculated from three independent datasets.

1. End-reaching times and termination times of phage RNAPs at wildtype terminators

| RNAP at terminator | τ_e_ (s) | *n* in replicated experiments | τ_t_ (s) | *n* in replicated experiments |
| --- | --- | --- | --- | --- |
| T7 RNAP at T7 *Tφ* | 7.5 ± 2.2 | 648 = 212 + 262 + 174 | 52.3 ± 13.4 | 749 = 235 + 307 + 207 |
| T7 RNAP at *t1* | 9.1 ± 0.9 | 131 = 49 + 41 + 41 | 46.5 ± 12.6 | 158 = 56 + 48 + 54 |
| T7 RNAP at *tR2* | 6.2 ± 0.4 | 269 = 108 + 88 + 73 | 75.5 ± 3.5 | 121 = 44 + 39 + 38 |
| T7 RNAP at *his* | 6.1 ± 6.0 | 96 = 27 + 37 + 32 | 50.8 ± 8.7 | 162 = 50 + 54 + 58 |
| T7 RNAP at *t500* | 1.9 ± 0.9 | 588 = 120 + 308 + 160 | 44.4 ± 7.6 | 84 = 17 + 46 + 21 |
| T7 RNAP at *t5* | 5.0 ± 4.6 | 577 = 186 + 236 + 155 | 69.6 ± 18.4 | 112 = 43 + 42 + 27 |
| T3 RNAP at T3 *Tφ* | 2.3 ± 1.0 | 283 = 104 + 107 + 72 | 14.1 ± 2.2 | 191 = 78 + 65 + 48 |
| SP6 RNAP at *t5* | 2.8 ± 0.7 | 50 = 22 + 14 + 14 | 20.6 ± 7.9 | 301 = 122 + 71 + 108 |

2. Termination times of phage RNAPs with RNAP supplements

| RNAP supplement | τ_t_ (s) median | τ_t_ (s) average | *n* in replicated experiments |
| --- | --- | --- | --- |
| 0 units/μl, 5% glycerol | 19.2 | 43.0 ± 6.7 | 344 = 102 + 116 + 126 |
| 5 units/μl, 5% glycerol | 13.6 | 37.0 ± 4.6 | 335 = 137 + 125 + 73 |
| 10 units/μl, 10% glycerol | 24.6 | 38.8 ± 3.9 | 231 = 76 + 84 + 71 |
